# Clinical and radiological imaging as prognostic predictors in COVID-19 patients

**DOI:** 10.1186/s43055-021-00470-9

**Published:** 2021-04-09

**Authors:** Maha Ibrahim Metwally, Mohammad Abd Alkhalik Basha, Mohamed M. A. Zaitoun, Housseini Mohamed Abdalla, Hanaa Abu Elazayem Nofal, Hamdy Hendawy, Esaraa Manajrah, Reham farid Hijazy, Loujain Akbazli, Ahmed Negida, Walid Mosallam

**Affiliations:** 1 Department of Radio-diagnosis, Faculty of Human Medicine, Zagazig University, Zagazig, Egypt; 2 Department of Radio-diagnosis, Faculty of Human Medicine, Suez Canal University, Esmaelia, Egypt; 3 Department of Community and Occupational Medicine, Faculty of Human Medicine, Zagazig University, Zagazig, Egypt; 4 Department of Intensive Care, Faculty of Human Medicine, Suez Canal University, Esmaelia, Egypt; 5 Faculty of Human Medicine, Suez Canal University, Esmaelia, Egypt; 6 Zagazig University Hospitals, Zagazig University, Zagazig, Egypt

**Keywords:** Novel coronavirus, COVID-19, Severity, chest CT, Fatal outcome, Oxygen saturation, Prognosis

## Abstract

**Background:**

Since the announcement of COVID-19 as a pandemic infection, several studies have been performed to discuss the clinical picture, laboratory finding, and imaging features of this disease. The aim of this study is to demarcate the imaging features of novel coronavirus infected pneumonia (NCIP) in different age groups and outline the relation between radiological aspect, including CT severity, and clinical aspect, including age, oxygen saturation, and fatal outcome. We implemented a prospective observational study enrolled 299 laboratory-confirmed COVID-19 patients (169 males and 130 females; age range = 2–91 years; mean age = 38.4 ± 17.2). All patients were submitted to chest CT with multi-planar reconstruction. The imaging features of NCIP in different age groups were described. The relations between CT severity and age, oxygen saturation, and fatal outcome were evaluated.

**Results:**

The most predominant CT features were bilateral (75.4%), posterior (66.3%), pleural-based (93.5%), lower lobe involvement (89.8%), and ground-glass opacity (94.7%). ROC curve analysis revealed that the optimal cutoff age that was highly exposed to moderate and severe stages of NCIP was 38 years old (AUC = 0.77, *p* < 0.001). NCIP was noted in 42.6% below 40-year-old age group compared to 84% above 40-year-old age group. The CT severity was significantly related to age and fatal outcome (*p* < 0.001). Anterior, centrilobular, hilar, apical, and middle lobe involvements had a significant relation to below 90% oxygen saturation. A significant negative correlation was found between CT severity and oxygen saturation (*r* = − 0.49, *p* < 0.001). Crazy-paving pattern, anterior aspect, hilar, centrilobular involvement, and moderate and severe stages had a statistically significant relation to higher mortality.

**Conclusion:**

The current study confirmed the value of CT as a prognostic predictor in NCIP through demonstration of the strong relation between CT severity and age, oxygen saturation, and the fatal outcome. In the era of COVID-19 pandemic, this study is considered to be an extension to other studies discussing chest CT features of COVID-19 in different age groups with demarcation of the relation of chest CT severity to different pattern and distribution of NCIP, age, oxygen saturation, and mortality rate.

## Background

On December 2019, an unidentified cause of pneumonia affected a cluster of a population in Wuhan, China [1]. On January 7, 2020, a novel strain of coronavirus was extracted from the patients’ respiratory tract secretions given the name of severe acute respiratory syndrome corona­virus 2 (SARS­CoV­2) [2, 3]. On March 11, 2020, WHO characterized COVID-19 as a pandemic [4]. On September 1, 2020, confirmed cases of COVID-19 reported to WHO were 25,298,875, including 847,602 deaths with the highest number of reported positive cases was in the USA, while the lowest number was in the Western Pacific [5].

Several studies reported a higher sensitivity of chest CT in comparison to real-time reverse transcriptase-polymerase chain reaction (RT-PCR) (98% vs. 71%, *p* < 0.001) [6]. RT-PCR test results take hours or even days to be available, which may delay COVID-19 patients’ triaging. Furthermore, RT-PCR tests are resource-constrained, which may limit its accessibility to all suspected patients. So, chest CT is considered now as a useful supplementary tool for RT-PCR [7]. Fang et al. [6] advocated using chest CT as a screening tool for SARS-CoV-2 for patients with clinical and epidemiologic features matched with COVID-19 infection even with negative RT-PCR.

Several observational studies, systematic reviews, and meta-analysis have been published discussing the clinical, laboratory, and imaging features of COVID-19 [8–13]. However, the relations between radiological features and the clinical aspects of COVID-19, particularly the relations between CT severity and age, oxygen saturation, and fatal outcome, need to undergo further investigations. These relations can confirm the prognostic value of chest CT in COVID-19 patients. Consequently, in this study, we tried to enrich the database with COVID-19 characteristics in Egypt by describing the imaging features in different age groups and underlining the relations between CT severity score, age, oxygen saturation, and fatal outcome.

## Methods

### Ethical considerations

The Zagazig University ethics committee approved this study (approval no. 6381; approved May 10, 2020). A written informed consent was obtained from all patients in this study. The study was conducted according to the ethical principles of the declaration of Helsinki. This manuscript was reported in adherence with the Strengthening the Reporting of Observational Studies in Epidemiology (STROBE statement guidelines).

### Study design and population

A prospective observational study was conducted between June 1, 2020 and September 25, 2020. A primary sample consisted of 328 laboratory-confirmed COVID-19 patients. All patients were confirmed positive by RT-PCR via nasopharyngeal swab. Exclusion criteria were (i) patients had CT examinations prior to hospital admission (*n* = 13), (ii) patients refused hospitalization (*n* = 9), and (iii) patients gave history of interstitial lung disease (*n* = 7). The exclusion process resulted in a final sample consisted of 299 patients. The patients were categorized into four distinct age groups: group 1; child (> 1–≤ 18 years), group 2; young adult (> 18–< 40 years), group 3; middle age (≥ 40–< 60 years), group 4; old age (≥ 60 years). The age, sex, oxygen saturation (≥ 95%, between 95 and 90%, and ≤ 90%), and survival/fatal outcome of all patients were documented.

### CT image acquisition

Non-contrast enhanced CT scans were obtained on the day of patients’ hospital admission using Aquillion lightning (Anon, Japan). The scanning area extended from the level of the upper thoracic inlet to the inferior level of the costophrenic angle with the following parameters: detector X collimation widths = 16 × 1/0.5 mm (adult/child); tube voltage = 120/80 kV (adult/child). The tube current was regulated by sure exposure 3D; AIDR (adaptive iterative dose reduction) 3D. The CT scans were acquired at the end of inspiration in co-operative patients. Reconstructed images were obtained with a slice thickness/interval = 1/1 mm. The reconstructed images were transferred to the workstation and picture archiving and communication systems (PACS) for post­processing.

### Image analysis

All CT images were interpreted in consensus by two radiologists (W.M and M.M with 12 and 8 years of chest imaging experience, respectively). The radiologists were blinded to the patients’ clinical data and laboratory results. The following features were evaluated on CT images: (a) distribution of the lung insult: (1) unilateral or bilateral, (2) involved axial aspect of the lung: posterior or anterior, (3) regional distribution within the lung: hilar, centrilobular, or pleural-based, (4) the most predominate distribution: centrilobular, pleural-based, or no dominate distribution, (5) lobar involvement: lower, upper, middle, or apical, and (6) presence of lower lobe predominance; (b) imaging pattern based on terms provided by Fleischner Society [14]: (1) ground glass opacity (GGO), (2) consolidation, (3) crazy paving, and (4) the most predominant imaging pattern: GGO, consolidation, or no predominate pattern; (c) other evaluated features, included presence of air bronchogram, halo sign, reversed halo sign, pleural effusion, septal thickening, subpleural line, parenchymal scaring (combination of: irregular subpleural lines, irregular parenchymal band, and traction bronchiectasis), fissure thickening, pleural sparing, lymph nodes enlargement, and any other coincidental finding; (d) CT severity score by subjective assessment of the extent of lung involvement based on personal experience: (1) subtle (< 5% ), (2) mild (5–< 30%), (3) moderate (30–< 60%), and (4) severe (≥ 60%).

### Statistical analysis

Analyses were done using SPSS version 20.0 (IBM, Armonk, NY). The normality of distribution was assessed using the Kolmogorov-Smirnov test. Normally distributed data were presented as mean and standard deviation (SD), and categorical data as frequency and percentage. The difference between groups was analyzed by using Chi-square and Fisher exact tests (for categorical data). The correlation was done to detect the linear relationship between two numerical variables using the Pearson correlation coefficient. ROC curve was used to detect the cutoff value of age highly exposed to be infected with COVID-19. *P* value ≤ 0.05 was considered significant.

## Results

### Patient demographic and clinical characteristics

The final analysis consisted of 299 RT-PCR positive patients (169 males and 130 females; age range = 2–91 years; mean age = 38.4 ± 17.2). Our patients were categorized into four distinct age groups. The demographic and clinical characteristics of the study population are shown in Table 1. All children, 91.7% of young age, and 74.1% of middle-age groups had ≥ 95% O_2_ saturation, whereas 52.8% of the old age group had ≤ 90% O_2_ saturation. Intubated patients were reported in the middle and old age groups (2.3%). The overall CFR was 13 (4.3%), with the highest CFR was in the old age group (16.7%).

**Table 1:** Demographic and clinical characteristics of the study population

Age group	No.	Age Mean + SD (range)	Sex		O_2_ saturation			Intubated patients	CFR
			Male	Female	≥ 95%	90–<95%	≤ 90%		
Child (> 1–≤ 18 years)	34	9.97 ± 4.5 (2-18)	23 (67.6)	11 (32.3)	34 (100)	0	0	0	0
Young age (> 18–< 40 years)	121	29.13 ± 5.8 (19-39)	68 (56.2)	53 (43.8)	111 (91.7)	6(5.0)	4 (3.3)	0	1 (0.8)
Middle age (≥ 40–< 60 years)	108	74.94 ± 5.9 (40-59)	64 (59.3)	44 (40.7)	80 (74.1)	13(12.0)	15 (13.9)	3 (2.8)	6 (5.6)
Old age (≥ 60 years)	36	67.55 ± 6.61 (60-91)	14 (38.9)	22 (61.1)	15(41.7)	2(5.6)	19 (52.8)	4 (11.1)	6 (16.7)
Total	299	38.37 ± 17.2 (2-91)	169 (56.5)	130 (43.5)	240 (80.3)	21(7.0)	38 (12.7)	7 (2.3)	13 (4.3)

*No* number, *SD* standard deviation, *CFR* case fatality rate

Unless otherwise indicated, data represent number of patients with percentage in parenthesis

### CT imaging features

**Fig. 1:**
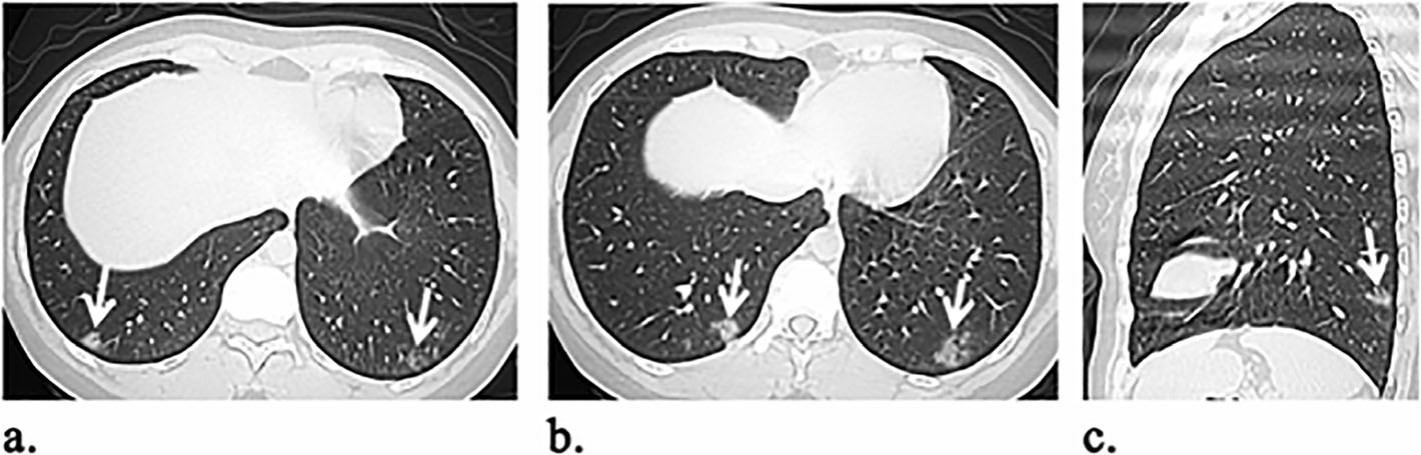
A 28-year-old female patient presented with significant myalgia and headache for 7 days. O_2_ saturation was 97%. **a**, **b** Axial and **c** sagittal unenhanced HRCT images reveal bilateral multifocal lower lobe lung infiltrations with small rounded patches of GGO and consolidation close to the pleura yet sparing it (white arrows)

**Fig. 2:**
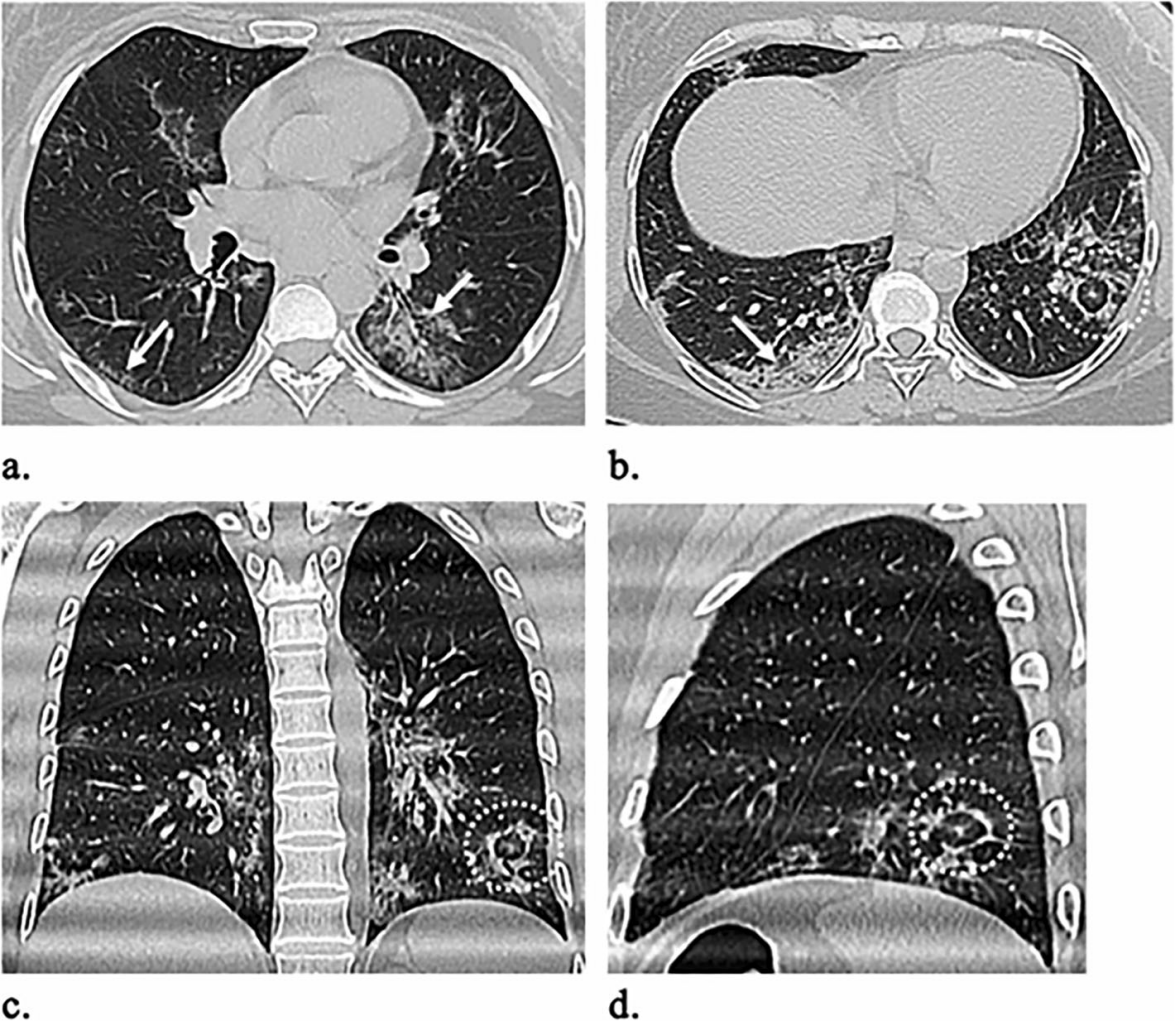
A 42-year-old female patient experienced high-grade fever and cough for 12 days. O_2_ saturation was 95%. **a**, **b** Axial, **c** coronal, and **d** sagittal unenhanced HRCT images reveal bilateral multifocal subpleural lower lobe predominant GGO and crazy paving (white arrows) with left lower lobe reversed Halo sign (dashed circle)

**Fig. 3:**
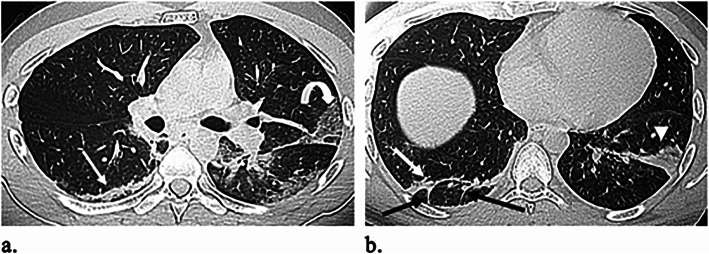
A 31-year-old male patient presented with a sore throat and dry cough. O_2_ saturation was 97%. **a**, **b** Axial unenhanced HRCT images reveal subpleural patches of consolidation (white arrowhead), crazy paving (curved arrow), subpleural lines (white arrows), and parenchyma bands (black arrows)

**Fig. 4:**
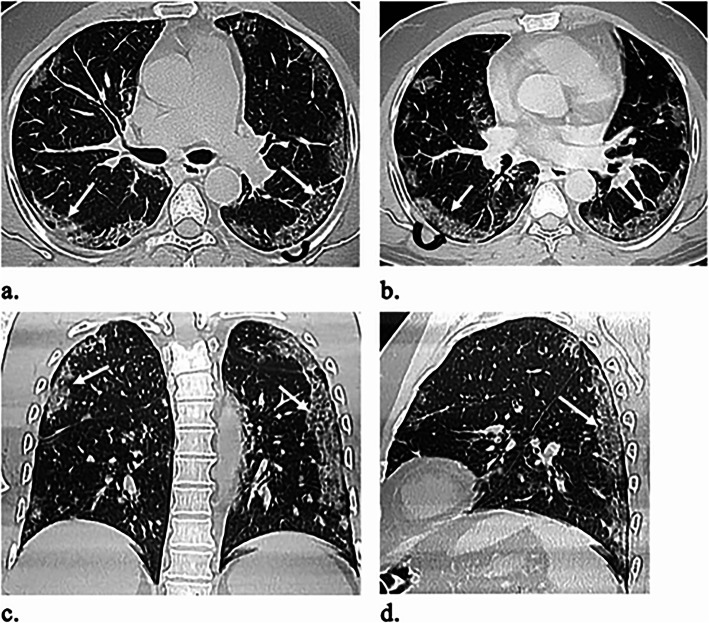
A 54-year-old male patient presented with 8 days history of breathlessness and cough. O_2_ saturation was 93%. **a**, **b** Axial, **c** coronal, and **d** sagittal unenhanced HRCT images show bilateral multifocal, mainly subpleural infiltrations with GGOs and crazy paving (white arrows). Subpleural sparing is noted (curved black arrows)

Table 2 outlines the CT imaging features in different age groups. Out of 299 patients, 187 (62.5%) had positive CT findings. Pleural-based was the most predominant axial distribution (67.9%). Lower lobe predominance was seen in 102 (54.5%) patients. GGO was the predominant pattern affecting the lung (58.8%) (Fig. 1). Other CT features, included air bronchogram (28.9%), halo sign (12.8%), reverse halo sign (3.2%) (Fig. 2), septal thickening (28.3%), subpleural lines (39.6%), parenchymal bands (43.3%) (Fig. 3), fissure irregularities (14%), significant scaring (10.2%), pleural sparing (39.6%) (Fig. 4), reactionary lymph nodes enlargement (21.1%), and no pleural effusion.

**Table 2:** CT imaging features of NCIP in different age groups

Age groups	Child (No = 4)	Young age (No = 62)	Middle age (No = 88)	Old age (No = 33)	Total (No = 187)
CT features					
Bilateralism					
Unilateral	2 (50)	21 (33.8)	17 (19.3)	6 (18.2)	46 (24.6)
Bilateral	2 (50)	41 (66.1)	71 (80.6)	27 (81.8%)	141 (75.4)
Anteroposterior distribution					
Anterior	1 (25)	29 (46.8)	61 (69.3)	25 (75.8)	116 (62)
Posterior	3 (75)	40 (64.5)	62 (70.5)	19 (57.6)	124 (66.3)
Axial distribution					
Hilar	0	24 (38.7)	46 (52.3)	21 (63.6)	91 (48.7)
Centrilobular	1 (25)	38 (61.2)	67 (76.)	28 (84.8)	134 (71.7)
Pleural based	3 (75)	56 (90.3)	85 (96.6)	31(93.9)	175 ( 93.5)
Predominant axial distribution					
Pleural based	3 (75)	43 (69.3)	60 (68.2)	21 (63.6)	127 (67.9)
Centrilobular	1 (25)	3 (4.8)	8 (9.1)	2 (6.3)	14 (7.5)
No specific	0	16 (25.8)	20 (22.7)	10 (30.3)	46 (24.6)
Lobar distribution					
Lower lobe	3 (75)	53 (85.5)	81 (92)	31 (93.9)	168 (89.8)
Middle lobe	0	27 (43.5)	58 (65.9)	27 (81.8)	112 (59.9)
Upper lobe	1 (25)	39 (62.9)	77 (87.5)	27 (81.8)	144 (7.7)
Apex	0	13 (20.9)	44 (50)	24 (72.7)	81 (43.3)
Lower lobe predominance	3 (75)	37 (59.6)	47 (53.4)	15 (45.5)	102 (54.5)
CT pattern					
GGO	4 (100)	60 (96.8)	82 (93.2)	31 (93.9)	177 (94.7)
Consolidation	0	31 (50)	55 (62.5)	19 (57.6)	105 (56.1)
Crazy-paving	0	16 (25.8)	35 (39.8)	14 (42.4)	66 (35.3)
Most predominant CT pattern					
GGO	4 (100)	36 (58.1)	50 (56.8)	20 (60.6)	110 (58.8)
Consolidation	0	12 (19.4)	22 (25)	4 (12.1)	38 (20.3)
No predominance	0	14 (22.6)	16 (18.2)	9 (27.3)	39 (20.9)

*No* number, *CT* computed tomography, *NCIP* novel coronavirus-infected pneumonia, *GGO* ground glass opacity

Data represent number of patients with percentage in parenthesis

### The relation between positive CT findings and age

**Fig. 5:**
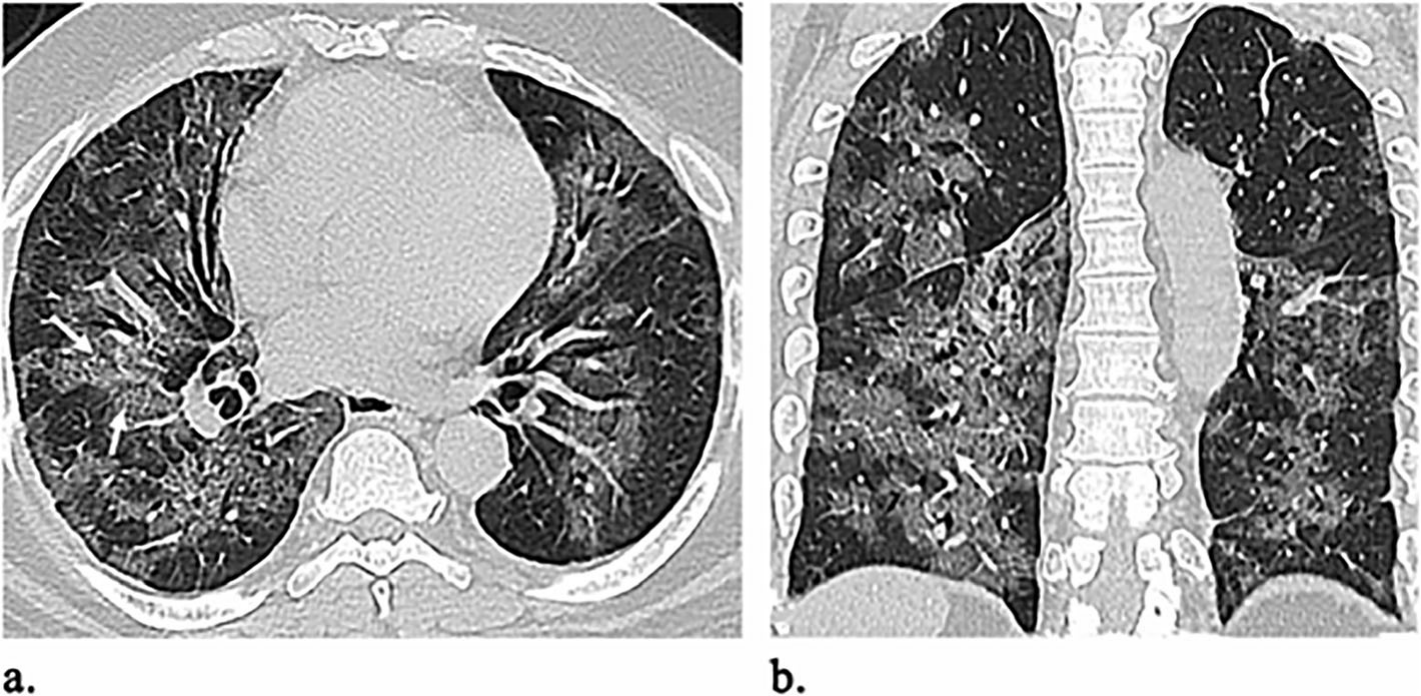
A 49-year-old female patient presented with epigastric pain, diarrhea, cough, and dyspnea. O_2_ saturation was 90%. **a** Axial and **b** coronal unenhanced HRCT images show bilateral upper and lower lobes extensive parenchymal involvement by GGO with intralobular septal thickening (white arrows) resembling crazy paving distributed in the hilar, centrilobular, and subpleural regions. No definite consolidations

On comparison between below and above 40-year-old age groups regarding positive CT findings, a strong significant relation was noted (*p* < 0.001) (Table 3). In below and above 40-year-old age groups, GGO was seen in 36.2% versus 63.8% (*p* = 0.29), consolidation was seen in 29.5% versus 70.5% (*p* = 0.062), crazy paving was seen in 25.8% versus 74.2% (*p* = 0.04) (Fig. 5), reversed halo sign was seen in 0% versus 100%, septal thickening was seen in 17% versus 83% (*p* = 0.001), subpleural line was seen in 25.7% versus 74.3% (*p* = 0.026), parenchymal band was seen in 25.9% versus 74.1% (*p* = 0.019), fissure irregularities were seen in 19.2% versus 80.8% (*p* = 0.062), significant scaring was seen in 21.1% versus 78.7 % (*p* = 0.17), pleural spacing was seen in 31.31% versus 68.8% (*p* = 0.02), and enlarged lymph nodes were seen in 17.9% versus 82.1 (*p* = 0.01).

**Table 3:** Relation between the incidence of NCIP and age

Age group	CT Positive	CT Negative	*P* value
Age <40 (No = 155)	66 (42.6)	89 (57.4)	<0.001
Age ≥ 40 (No = 144)	121 (84.02)	23 (15.97)	

*No* number, *CT* computed tomography, *NCIP* novel coronavirus-infected pneumonia

Date represent the number of patients with percentage in parenthesis

### The relation between CT severity score and age

A significant relation was noted between age and CT severity score (*p* ˂ 0.001) (Table 4). The ROC curve analysis revealed that > 38-year-old was the optimal cutoff value of age that was highly exposed to develop moderate and severe stages of NCIP (AUC = 0.77, 95% CI = 0.72–0.82 *p* < 0.001).

**Table 4:** Relation between CT severity score and age

Age group (No)	CT severity				*P* value
	Subtle	Mild	Moderate	Severe	
Child (4)	3 (75)	1 (25)	–	–	<0.001
Young age (62)	27 (43.5 )	17 (27.4)	14 (22.6)	4 (6.5)	
Middle age (88)	13 (14.7)	30 (34)	32 (36.6)	13 (14.7)	
Old age (33)	3 (9.1)	8 (24.2)	9 (27.3)	13 (39.4)	

*CT* computed tomography, *NCIP* novel coronavirus-infected pneumonia

Date represent the number of patients with percentage in parenthesis

### The relation between CT severity score and CT features

All lobar and regional lung distribution had nearly the same impact on CT severity score except posterior lung affection (*p* = 0.57) (Table 5).

**Table 5:** Relation between CT severity score and CT features

CT features (No)	CT severity				*P*-value
	Subtle	Mild	Moderate	Severe	
Bilateralism					<0.001
Unilateral (46)	31 (67.4)	12 (26.1)	3 (6.5)	0	
Bilateral (141)	15 (10.6 )	44 (31.2)	52 (36.9)	30 (21.3)	
Anteroposterior distribution					
Anterior					
Negative (70)	33 (47.1)	30 (42.9)	7 (10)	0	<;0.001
Positive (116)	13 ( 11.2)	26 (22.4)	47 (40.5)	30 (25.9)	
Posterior					
Negative (63)	20 (31.7)	11 (17.5)	21 (33.3)	11 (17.5)	0.57
Positive (124)	26 (21.0)	45 (36.3)	34 (27.4)	19 (15.3)	
Axial distribution					
Hilar					
Negative (96)	38 (39.6)	37 (38.5)	21 (21.9)	0	<0.001
Positive (91)	8 (8.8)	19 (20.9)	34(37.4)	30(33	
Centrilobular					
Negative (53)	30 (56.6)	15(28.3)	8 (15.1)	0	<0.001
Positive (134)	16 (11.9)	41 (30.6)	47 (35.1)	30 (22.4)	
Pleural based					
Negative (12)	10 (83.3)	2 (16.6)	0	0	<0.001
Positive (175)	37 (21.1)	53 (30.3)	55 (31.4)	30 (17.1)	
Lobar distribution					
Lower lobe					
Negative (19)	12 (63.2)	5 (26.3)	2 (10.5 )	0	<0.001
Positive (168)	34 (20.2)	51 (30.4)	53 (31.5)	30 (17.9)	
Middle lobe					
Negative (75)	39 (52)	30 (40)	6 (8)	0	<0.001
Positive (112)	7 (6.2)	26 (23.2)	49 (43.8)	30 (26.8)	
Upper lobe					
Negative (43)	29 (67.4 )	13 (30.2)	1 (2.3)	0	<0.001
Positive (144)	17 (11.8)	43 (29.9)	54 (37.5)	30 (20.8)	
Apex					
Negative (106)	46 (43.3)	42 (39.6)	17 (16.3)	1 (1)	<0.001
Positive (81)	1 (1.2)	15 (18.5)	38 (46.9)	27 (33.3)	
CT pattern					
GGO					
Negative (10)	1 (10.0)	7 (70)	1 (10)	1 (10)	0.043
Positive (177)	45 (25.4)	49 (27.7)	54 (30.5)	29 (16.4)	
Consolidation					
Negative (82)	38 (46.3)	27 (32.9)	11 (13.4)	6 (7.3)	<0.001
Positive (105)	8 (7.6)	29 (27.6)	44 (41.9)	24 (22.9)	
Crazy-paving					
Negative (121)	40 (33.1)	41 (33.9)	31 (25.6)	9 (7.4)	<0.001
Positive (66)	6 (9.1)	15 (22.7)	24 (36.4)	21 (31.8)	

*CT* computed tomography, *GGO* ground glass opacity

Date represent the number of patients with percentage in parenthesis

### The relation between CT features, severity score, and oxygen saturation

**Fig. 6:**
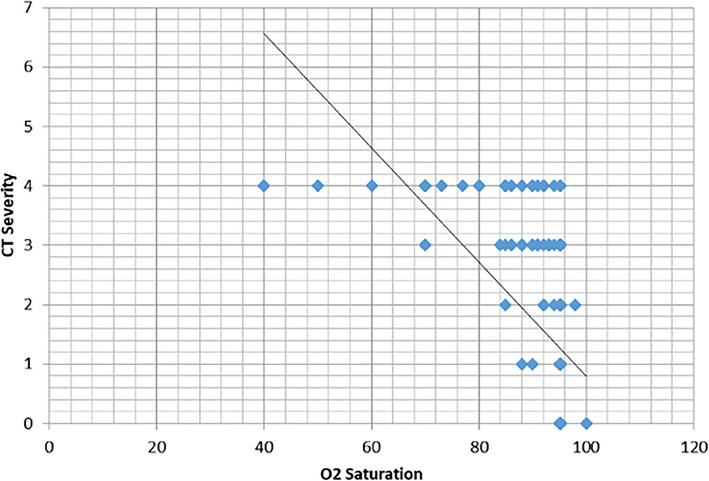
Correlation diagram shows a significant negative correlation between CT severity and oxygen saturation (*r* = − 0.49, *p* < 0.001)

Most CT features had a significant relation with oxygen saturation except for pleural-based lower lobe distribution and GGO (Table 6). A significant negative correlation was noted between CT severity and oxygen saturation (*r* = − 0.49, *p* ˂ 0.001) (Fig. 6). A significant relation was noted on comparing moderate and severe stages of the CT severity score with oxygen saturation, with 73.3% of severe cases developed ≤ 90% oxygen saturation (*p* < 0.001).

**Table 6:** Relation between CT features, severity score, and oxygen saturation

CT features (No)	O_2_ saturation		*P* value
	>90%	≤ 90%	
Bilateralism			0.007
Unilateral (46)	43 (93.3)	3 (6.5)	
Bilateral (141)	106 (75.2)	35 (24.8)	
Anteroposterior distribution			
Anterior			
Negative (70)	67 (95.7)	3 (4.3)	<0.001
Positive (116)	81 (69.8)	35 (30.2)	
Posterior			
Negative (63)	42 (66.7)	21 (33.3 )	0.002
Positive (124)	107 (86.3)	17 (13.7)	
Axial distribution			
Hilar			
Negative (96)	90 (90.3)	6 (6.2)	<0.001
Positive (91)	59 (64.8)	32 (35.2)	
Centrilobular			
Negative (53)	51 (96.2)	2 (3.8)	<0.001
Positive (134)	98 (73.1)	36 (26.9)	
Pleural based			
Negative (12)	11 (91.6)	1 (8.3)	0.33
Positive (175)	138 (78.9)	37 (21.1)	
Lobar distribution			
Lower lobe			
Negative (19)	18 (94.7)	1 (5.3)	0.085
Positive (168)	131 (78)	37 (22)	
Middle lobe			<0.001
Negative (75)	71 (94.7)	4 (5.3)	
Positive (112)	78 (69.6)	34 (30.4)	
Upper lobe			0.004
Negative (43)	41 (95.3)	2 (4.7)	
Positive (144)	108 (75)	36 (25)	
Apex			<0.001
Negative (106)	98 (92.4)	8 (7.7)	
Positive (81)	52 (64.2)	29 (35.8)	
CT pattern			
GGO			
Negative (10)	10 (100)	0	0.2
Positive (177)	139 (78.5)	38 (21.5)	
Consolidation			
Negative (82)	71 (86.6)	11 (13.4)	0.038
Positive (105)	78 (74.3)	27 (25.7)	
Crazy-paving			
Negative (121)	106 (87.6)	15 (12.4)	<0.001
Positive (66)	43 (65.2)	23 (34.8)	

*CT* computed tomography, *GGO* ground glass opacity

Date represent the number of patients with percentage in parenthesis

### The relation between CT features, severity score, and fatal outcome

A significant relation was statistically confirmed between CT severity score and fatal outcome manifested by increasing the number of deaths in moderate and severe stages (*p* < 0.001). A significant relation was noted between the apex and hilar distribution and fatal outcome (*p* < 0.001) (Table 7).

**Table 7:** Relation between CT features, severity score, and fatal/survival outcome

CT features (No)	Fate		*P* value
	Death	Survival	
Bilateralism			0.03
Unilateral (46)	0	46 (100)	
Bilateral (141)	13 (9.8)	128 (90.8)	
Anteroposterior distribution			
Anterior			0.004
Negative (70)	0	70 (100)	
Positive (116)	13 (11.1)	104 (88.8)	
Posterior			0.08
Negative (63)	4 (6.3)	59 (93.7)	
Positive (124)	9 (7.3)	115 (92.7)	
Axial distribution			
Hilar			0.001
Negative (96)	1 (1)	95 (99)	
Positive (91)	12 (13.2)	79 (86.2)	
Centrilobular			0.019
Negative (53)	0	53 (100)	
Positive (134)	13 (9.7)	121 (90.3)	
Pleural based			0.3
Negative (12)	12 (100)	12 (100)	
Positive (175)	162 (92.6)	162 (92.6)	
Lobar distribution			
Lower lobe			0.2
Negative (19)	0	19 (100)	
Positive (168)	13 (7.7)	155 (92.3)	
Middle lobe			0.002
Negative (75)	0	75 (100)	
Positive (112)	13 (11.6)	99 (88.4)	
Upper lobe			0.04
Negative (43)	0	43 (100)	
Positive (144)	13 (9)	131 (91)	
Apex			<0.001
Negative (106)	0	105 (99.1)	
Positive (81)	13 (9)	69 (85.2)	
CT pattern			
GGO			0.37
Negative (10)	0	10 (100)	
Positive (177)	13 (7.3)	164 (92.3)	
Consolidation			0.28
Negative (82)	4 (4.9)	78 (95.1)	
Positive (105)	9 (8.6)	96 (91.4)	
Crazy-paving			0.008
Negative (121)	4 (3.3)	117 (96.7)	
Positive (66)	9 (13.6)	57 (86.4)	
CT severity			<0.001
Subtle (46)	0	46 (100)	
Mild (56)	0	56 (100)	
Moderate (55)	2 (3.6)	53 (96.4)	
Severe (30)	11 (36.7)	19 (63.3)	

*CT* computed tomography, *GGO* ground glass opacity

Date represent the number of patients with percentage in parenthesis

## Discussion

Our results demonstrated that the incidence of NCIP was statistically higher in the above 40-year-old age group with progressively increasing the CT severity score with advanced age. The most susceptible age to develop moderate and severe stages of NCIP was > 38 years old. The severe stage of NCIP included the highest percentage of patients with ≤ 90% oxygen saturation and with fatal outcome.

In our study, the CFR was 4.3%. Previous studies [13, 15–17] showed CFR ranging between 4.3 and 15%. This value may be attributed to the presence of variable stages of the disease severity in addition to variable age groups in our admitted patients. In our research, the CFR was age-linked, increased from 0.8% in the young age group to 16.6% in the old age group. This finding is endorsing with Ferguson et al. [18], who reported that CFR increased with age from < 0.6 to 2.2% at 60 years old and reached over 9.3% at 80 years old.

Keeping with the results of previous studies [19–21], we found that the most predominant CT features in NCIP were bilateral, posterior predominance, pleural-based, lower lobe involvement, and GGO. The other CT findings, including bronchiectasis, interlobular septal thickening, subpleural involvement, and pleural thickening, were reported with different percentages among studies [22–25]. Pleural effusion, lymphadenopathy, CT halo sign, pericardial effusion, cavitation, and pneumothorax were reported less commonly or in rare cases [26, 27]. We confirmed Shi et al. [28], who stated the absence of tree­in­bud, cavitations, masses, and calcifications, suggesting bacterial or chronic infections.

Chest CT findings of NCIP regarding different age groups were described in two studies [26, 27]. Song et al. [26] divided the studied population into above and below 50 years and found severe lung involvement with consolidations in above 50-year-old patients, while others younger than 50 years had more GGOs. Another study [27] classified 72 symptomatic patients into above and below 60 years and documented severe multilobar affection in older patients (71.4% vs. 36.4%, *p* = 0.009) with pleural thickening and subpleural line (71.4% vs. 40.9%, and 50.0% vs. 25.0%, *p* = 0.011 and 0.030, respectively). However, in our study, GGO and consolidation were observed more in the above 40-year-old group, but with no statistically significant relation (*p* = 0.29 and 0.06, respectively).

A significant relation was proved between CT severity and older age groups. Bilateral and anterior aspect involvement of the lung had an obvious relation with the severe stage. However, no specific pattern or distribution had a predilection to the severe stage more than the others.

Resting oxygen saturation < 95% is considered abnormal [29]. Karimi et al. [30] established oxygen saturation < 93% with oxygen assistance or < 90% at room air as a sign of severe pneumonia. Other studies also depended on oxygen saturation ≤ 90% by pulse oximetry to define hypoxia in the pneumonia severity index score [31, 32]. According to Andrea et al. [33], mixed GGO and consolidation was noted in critically ill patient with lower oxygen saturation while GGO only was noted in non-critically ill patient with higher oxygen saturation. In our study, GGO and consolidation had non-significant relation to oxygen saturation while crazing–paving was the main pattern associated with below 90 % oxygen saturation. Up to our knowledge, no study has correlated the distribution of NCIP and oxygen saturation. In our study anterior, centrilobular, hilar, apical, and middle lobe involvements had a significant relation to below 90% oxygen saturation. A significant negative correlation between CT severity and oxygen saturation was statistically proved.

In our study, crazy-paving, anterior aspect, hilar, centrilobular involvement, and moderate and severe stages had a statistically significant relation to higher mortality. The relation between crazy-paving pattern and higher mortality may be explained by the autopsy results from cases with crazing–paving that revealed diffuse alveolar damage with different stages of inflammation and fibrosis [34].

Finally, the relations mentioned above approve the prognostic value of CT in NCIP, which may predict the outcome of the COVID-19 patients and alter the management strategy in a trial to decrease the disease morbidity and mortality.

We encountered limitations in our study. First, chest CT was done for all patients on the day of hospital admission regardless of the onset of symptoms. Second, we did not acquire pulmonary CT angiography to evaluate the possibility of thromboembolic lung affection. Third, we did not take laboratory findings of our patients into consideration. Lastly, we did not consider the impact of comorbidity factors on CT severity, oxygen saturation, and fatal outcome.

## Conclusion

In COVID-19 patients, CT severity is age-related, and most severe cases occur in the old age group. The prognostic value of CT in NCIP emits from the confirmed relations between CT imaging features, CT severity, oxygen saturation, and outcome of the patient. In consensus, CT could be considered the main determining factor in the management strategy of COVID-19 patients.

## Data Availability

All data are available on a software system owned by each of the authors and the corresponding author has the authority to respond if there is any query.

## Abbreviations

COVID: Coronavirus disease
SARS-CoV-2: Severe acute respiratory syndrome coronavirus 2
NCIP: Novel coronavirus infected pneumonia
CFR: Case fatality rate
GGO: Ground glass opacity
RT-PCR: Real-time reverse transcriptase-polymerase chain reaction
ROC: Receiver operating characteristic
